# Change in Electric Contact Resistance of Low-Voltage Relays Affected by Fault Current

**DOI:** 10.3390/ma12132166

**Published:** 2019-07-05

**Authors:** Andrzej Ksiazkiewicz, Grzegorz Dombek, Karol Nowak

**Affiliations:** Institute of Electric Power Engineering, Poznan University of Technology, Piotrowo 3A, 60965 Poznan, Poland

**Keywords:** relays, contact materials, electric contact resistance, contact welding

## Abstract

Contact resistance is an important maintenance parameter for electromagnetic switches, including low-voltage relays. The flow of significant current through electric contacts may influence the contact surface and thus the value of the electric contact resistance (ECR). The change in ECR is influenced not only by the value of current but also by the current phase. Therefore, the impact of the switching short-circuit current’s phase on the ECR was analyzed in this paper. Significant changes in the resistance after each switching cycle were observed. The ECR decreased significantly after each make operation, and a correlation with current amplitude, total let-through energy, and short-circuit time was not observed.

## 1. Introduction

Low-voltage relays are commonly used to connect circuits with moderate switching currents. They have found an application mainly as executive elements in building automation systems [1,2] that are becoming more and more popular, as well as in programmable controllers [3,4]. The relays available on the market differ not only in technical parameters, but also in construction and purpose.

An analysis of the literature on relays shows a certain research gap in the field of low- and medium-current AC switches. Research presented in referenced literature focuses either on different materials, like Ag-W, or higher test currents, over 1 kA, and contact force up to 50 N. It seldom touches upon the issues related to lower static contact force, in the range of centinewtons together with voltages up to 230 V_AC_ and test currents reaching 300 A.

**Figure 1:**
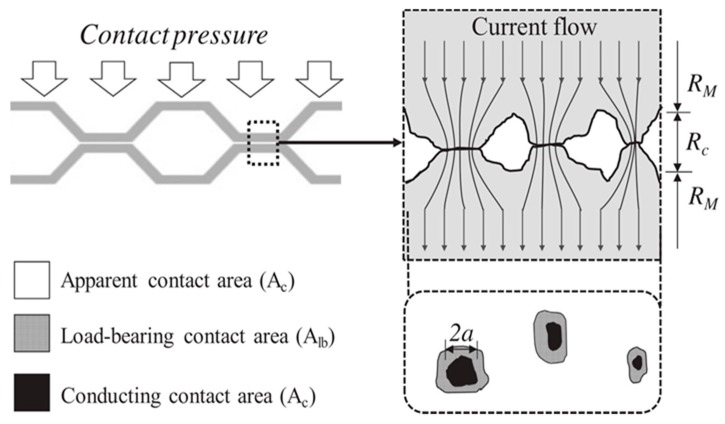
Electrical surface contact model with asperities and conducting contact area [8].

In order to describe contact resistance, it is common to apply a one-point model with ellipsoidal, equipotential dimensioning currents [5]. There are also attempts to make new models to describe the value of contact resistance for two conducting surfaces [6]. The microstructure of a constant surface is presented on Figure 1. The conducting contact area *A_c_* is much smaller than the nominal (apparent) surface *A_C_*. The difference is significant and the actual contact area may constitute about 5% of the apparent contact surface [7,8].

The contact resistance of an electro-energetic switchgear is its significant parameter of use. It is important that the resistance in the maintenance period of a relay reach the smallest possible values and, at the same time, that it not change over time. Its value influences the acceptable working load of a relay which is related to its heating. The value of contact resistance depends on the shape and resistance of the thin film layer [5]. Resistance of the thin film layer *R_n_* is difficult to establish analytically, as it depends on many factors, sometimes random, including ambient temperature, humidity, and contact material. Shape resistance *R_k_* mainly depends on contact material and the clamping force of contacts.

The value of the relay’s contact resistance is influenced by the material being used. The contact surface may be made of pure metals, including copper, silver, gold, platinum, palladium, wolfram, or molybdenum. However, alloys and sinters are more often used, such as silver-copper, silver-cadmium, silver-palladium, silver-cadmium oxide, silver-wolfram, silver-nickel, and silver-tin oxide [9]. The contact surface may be covered with an additional layer of material in order to enhance its properties (e.g., resistance to material transfer). Coatings made of tin, silver, or gold are also applied. The coating of a contact point with a layer of tin leads to a minimal increase of contact resistance in comparison to a material that is not coated. The layer of silver causes the reverse effect of decreasing the value of transition contact resistance [10].

Presently, the most common contact materials for low-voltage relays of alternating currents of average power are sinters of silver with nickel (AgNi), cadmium oxide (AgCdO), and tin oxide (AgSnO_2_). Materials that are made of silver-metal or silver-metal oxide typically present higher resistance to welding [11].

The frequency of occurrence and the force of the welding of contacts increase proportionally to the value of amperage of the electric arc, whereas the time of ignition in the arc does not have the same impact [12]. The force of the welding of contacts does not show any dependence on the static force of clamping between contact surfaces. However, it is dependent on the travelling pace of the moveable contact. These considerations are adequate mainly for those contacts made of pure silver [12]. Some contact materials present a higher probability of welding than others. If a contact material is characterized by a high tendency to welding, then the joints will be strong. In this regard, pure silver has the worst properties, showing a higher tendency to the welding of contacts [13], and it is one of the reasons why it is not used as contact material. Slightly better parameters are presented by AgCdO. It has a lower tendency to welding but it is of higher resistance. Contact materials such as AgSnO_2_ and AgNi, used for the analyzed relays, are characterized by similar properties in terms of welding and the resistance to tearing apart [14]. In this case, the first one creates stronger welds, but it proves that it has a lower tendency for their occurrence.

This article describes the influence of the short-circuit current phase on the change in electric contact resistance. The contact materials tested were AgNi, AgCdO, and AgSnO_2_. The AgSnO_2_ was tested in two variations. For the first one, contact rivets were made using the internal oxidation process and are referred to in the article as simply AgSnO_2_. For the second one, the rivet was designed to withstand higher inrush currents (up to 80 A for 20 ms) and are referred to in the paper as AgSnO_2_ P. In previous work [15], the experiments were carried out only for current switching at phase equal to zero and the AgCdO material was not tested. The results of this study are helpful in assessing contact materials used in low-voltage relays as they indicate how the contact resistance may change while switching fault current. This is an important factor for the long-term exploitation of relays used in modern electrical installations.

## 2. State of the Art

Relays intended for connecting the electrical load are prone to some disadvantageous switching phenomena. These phenomena may include the occurrence of overload currents and short-circuit currents that, as they flow through the relay contacts, can affect their surface condition and, consequently, the value of the contact resistance, which is an important operational parameter of the relay. This situation may also lead to a shortening of the relays’ maintenance time or, in extreme cases, to their complete destruction. Moreover, long-term exposure to higher temperature may also lead to a relay’s degradation, for example, its contact resistance and opening and closing times [16,17]. The corrosion film results in an increase in contact resistance and thus a decline in the contact performance.

The problems associated with low-voltage relay contacts are one of the research studies undertaken at several research centers in the world. The literature presents the test results of contacts made of various materials [12,13,18]. These tests are carried out both under normal operating conditions as well as under conditions of specific exposures (e.g., short circuits). Morin et al. [13], Neuhaus et al. [12], and Doublet et al. [18], who independently undertook work for similar contact materials (AgNi, AgCdO, and AgSnO_2_), focused their research on low-current circuits of direct current and small amperage. As shown by Morin et al. [13], each make operation results in contact material transfer (high for AgCdO and lower for AgNi and AgSnO_2_) and a high welding tendency for AgCdO, lower for the latter two. According to Neuhaus et al. [12], the welding force is hardly influenced by the static contact force. Supply voltage values sufficiently higher than the minimum arc voltage cause stable bounce arcs lasting the total bounce period. This is the case in the presented research, as the supply voltage is higher than the minimum arc voltage. In turn, the publication by Doublet et al. [18] states that AgSnO_2_ gives the best performance under short arcs as compared to Ag and AgNi. It presents a low welding and erosion tendency for short arcs with a higher erosion with longer arcs. However, these studies focus only on low-voltage (< 50 V) DC circuits.

In the literature related to relays, there are also papers about the processes of making circuits of alternating current having an average voltage and an amperage of several kA [19,20,21]. The operation of making significant currents may lead to contact bounces. Together with the increase of values for the contacting current’s amperage, there is the increase in the mass loss of the contact rivet [11,21,22,23].

## 3. Experimental Section

### 3.1. Materials Used and Their Characteristics

The tests considered the following contact materials: AgNi, AgCdO, as well as AgSnO_2_ (bimetal rivet) and AgSnO2 P (single metal rivet). Each of them was composed of 90% silver and a 10% addition of nickel, cadmium oxide, and tin oxide, respectively. Bimetal rivets are mainly made using powder metallurgy technology or, in the case of metal oxides, using so-called internal oxidation.

Single metal rivets are mainly produced from wires made of a certain contact material and their shape is obtained through the cold forging process. Selected properties of the contact materials used in the tests are presented in Table 1.

### 3.2. Testing Circuit Diagram

**Figure 2:**
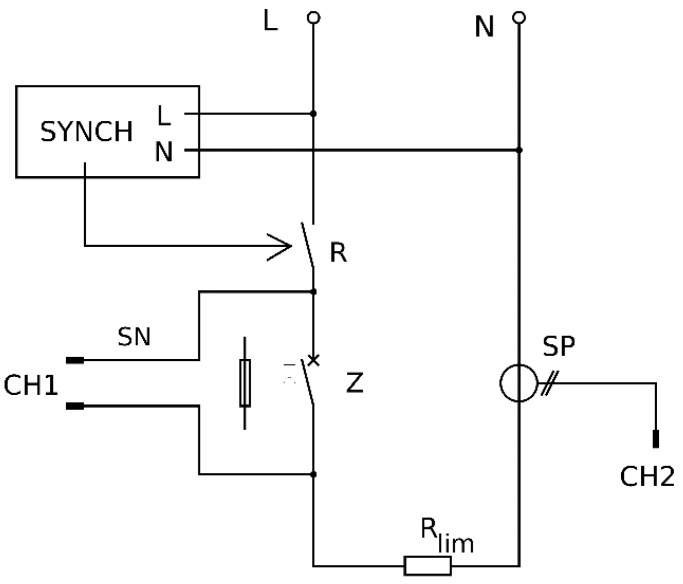
Testing circuit diagram: *R*, tested relay; *Z*, protection of the circuit (installation switch or fuse); *R_lim_*, limiting resistor; SYNCH, synchronizing device; SP, current probe; SN, voltage probe; CH1, CH2, oscilloscope channels [15].

**Figure 3:**
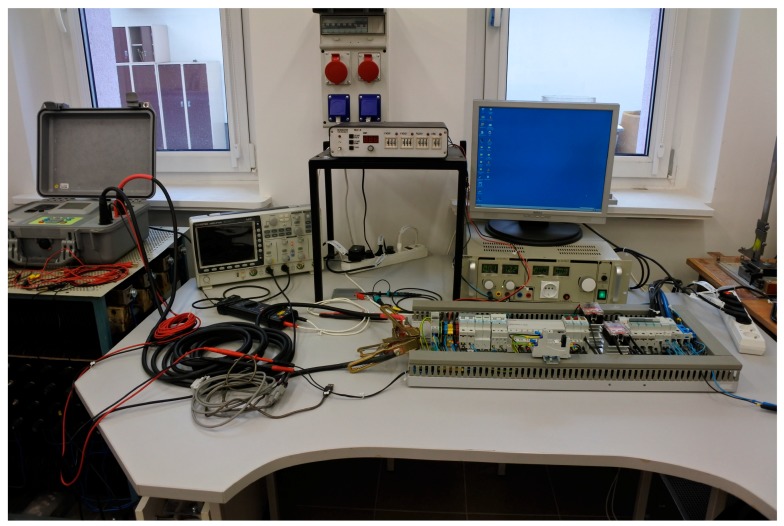
Photograph of the test bench used in the study.

The circuit diagram for a testing circuit is presented in Figure 2 and the test bench in Figure 3. The circuit is supplied directly from the power network having a low voltage of 230 VAC.

The circuit is protected against short circuits and overloads with installation switches of rated operating current 16 A and characteristics B, C, or D, and also with a fuse for general use gG 16.

For each contact material, a single test was conducted for every protection device. That resulted in four contact resistance values, for each contact material, before and after the test. A single test was executed for each tested relay, as each test had to been concluded on a new set of contacts. Four relays were tested for each contact material, and different protection device as mentioned above, which resulted in sixteen test trials. Due to the synchronizing device used at the moment of switching on the relay to the selected voltage phase, it was possible to obtain the repeatability of testing conditions.

For the short-circuit current phase, two extreme cases were selected—at the transition of the voltage through zero and at the moment when the supplying voltage reached a maximum value. Since the circuit was of resistant nature (power ratio *j* ≈ 0.99), the current in the circuit was in phase of supply voltage. The results obtained for the switching-on at zero of the supplying current for contacts made of AgNi and AgSnO_2_ have already been presented in [15]. The expected amperage of short-circuit current was limited with a resistor *R_lim_* (resistance value set is 0.729 Ω) to the value of 320 A (*I_m_* = 453 A). The average top value of a short-circuit current for tests of switching-on at zero of the supplying current equaled 421 A and, for switching-on at the maximum value of voltage, an average amplitude of current reached 457 A. The average value reached during the test may have exceeded the *I_m_* value because of power supply voltage fluctuations as the test stand was supplied directly from the public power network. However, the difference is less than 0.88% and can be omitted from the discussion. Because of the protection device’s limiting activity, the maximum value of the current for switching-on at zero was lower than the value of amplitude of the expected current.

The instantaneous value of the current in circuit may be described using Equation (1). The test circuit has very low induction L, so the power ratio is almost equal to one, that is, *φ* = 0. The set stand is powered from the public network, so the pulsation ω is equal to 314 rad·s^−1^ and the load is pure ohmic, that is, *ψ* = 0. For the two selected cases, when the voltage reached zero and maximum, it can be simplified as follows. For the first case *φ* = 0 and the exponential part of Equation (1) is equal to zero, the result is presented in Equation (2). For the second case *φ* = π/2 and the exponential part is also zero, the result is presented in Equation (3).

(1) $$i=I_{m}\left[\sin\left(\omega t+\psi +\varphi \right)-e^{-\frac{R}{L}}\sin\left(\psi -\varphi \right)\right]$$

(2) $$i=I_{m}\sin\left(\omega t\right)$$

(3) $$i=I_{m}\cos\left(\omega t\right)$$

**Figure 4:**
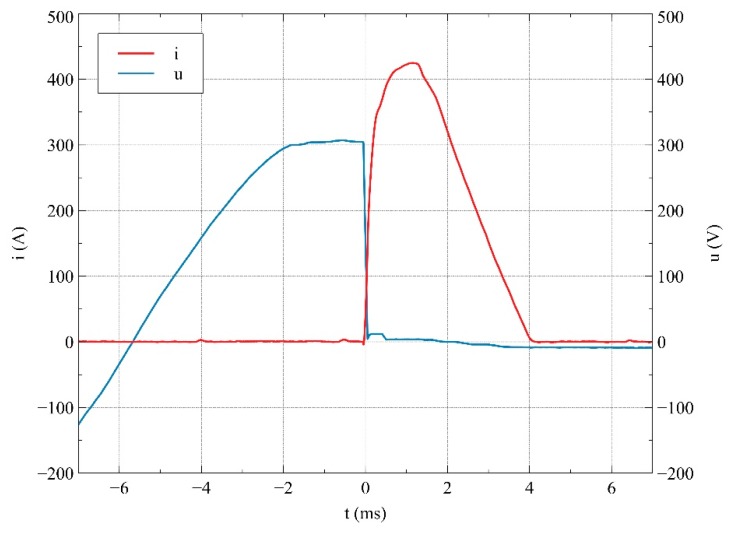
Current and voltage waveforms for a selected test for voltage phase *Φ* = 90°; contact material is AgSnO_2_ P.

The oscillogram of current and voltage between the contacts was registered using an oscilloscope GW Instek GDS-3154 (GW Instek, Taiwan), with a current probe and a voltage probe. An example of the oscillogram presenting the current in the circuit and the voltage between the contacts of a relay at short-circuit current phase of supplying voltage *Φ* = 90°, for contact material AgSnO_2_ P, is shown in Figure 4. Since the current flow lasted less than 7 ms, the heating of the contact has been omitted from the discussion.

The relays were switched to the circuit through a dedicated connecting socket. The measurement of resistance was performed with Kelvin’s 4-wire method using a meter for small resistance, MI3252 (Metrel, Horjul, Slovenia). In order to eliminate a measuring error of the expected value of contact resistance, the correction value was introduced which considered the resistance of the current paths for both the socket itself and a relay. Measuring current was 10 A with a reading accuracy of ± 0.25% and with a range of 2000 mΩ to 199,999 mΩ. The contact force for each contact material is presented in Table 2. Two different constructions of relays were used, one with AgNi AgSnO_2_ and AgSnO_2_ P materials, and the other with AgCdO. Those two relay models distinguished themselves with different closing mechanisms that led to varied contact forces.

## 4. Results and Discussion

The tests analyzed four models of relays with contacts made of materials presented in Section 3.1. Each relay underwent a single connecting trial. Before and after the trial of making a short-circuit current, the resistance value was measured. The trials were performed for different, commonly used, protection devices against short circuits and overloads.

Table 3 presents the mean values of the time period for short circuit *t_z_*, Joule’s integral *i*^2^*t*, and the maximum of a short-circuit current *i_m_* at switching of a short-circuit current, depending on the applied protection. It can be observed that the shortest periods of short circuits (the shortest operating time of the protection device) are seen for protection devices when the circuit was switched at the moment of maximum of supply voltage. The differences between time periods are small and result from the properties of certain devices. The time of the short circuit *t_z_* was calculated in a manner presented in [24]. Short-circuit time *t_z_* is calculated from the moment when the contacts are closed to the moment when the current is switched off by the protection device; therefore, it represents the total time for which the contact is conducting electrical current. For energy *i*^2^*t*, transported through particular switches during a short circuit occurring at switching at zero of voltage, the sequence of values is as follows (from the lowest to the highest value): fuse gG 16, switch B16, C16, and D16. There is a visible (more than 1.5 times) difference between the lowest and the highest value. Besides the case of a fuse gG 16, at switching at zero of voltage, the value of Joule’s integral is lower for trials of switching at maximum of supply voltage. For mean values of maximal current, there are small differences between the applied protection device. There are no visible relations between the applied protection device and the power transported during a short circuit and the value of contact resistance after the trial.

**Figure 5:**
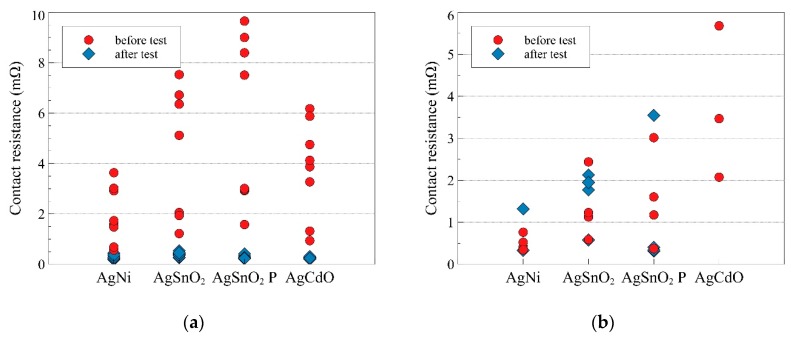
Change in contact resistance under the influence of switching of a short-circuit current at voltage phase: (**a**) *Φ* = 0°, (**b**) *Φ* = 90°; *P*, relay with full rivets.

The changes in the values of contact resistance before and after the trial, for the two above-mentioned cases of switching the testing circuit, are presented in Figure 5. Mean values of this resistance are presented in Table 4. First, there is the case of closing the relay contacts at zero value of supply voltage. The values of contact resistance before performing the trial are different for each of the analyzed materials. Comparing the mean values for them before performing the trial, it can be observed that the highest value characterizes the contacts which are made of AgSnO_2_ P, then, in decreasing order, they are AgSnO_2_, AgCdO, and AgNi. After the trial of switching the short-circuit current, the contact resistance is changed. For each material, there was a significant decrease of its value. The mean value of contact resistance for all trials equals 0.3053 mΩ and does not show a significant difference for particular materials. For each of the trials, no welding of contacts was observed.

In the case of closing the contacts of a relay at maximal value of supply current, the value of contact resistance before the trial (for AgNi, AgSnO_2_, and AgSnO_2_ P) is lower than in the previous case, when the make operation was made at zero current value. The difference exists despite the fact that there is no selection of particular relay items; they were selected randomly, and there is no initial surface treatment. For contacts made of AgSnO_2_, the value of the contact resistance increased after the performance of one of the trials. It was the only such case. The most significant change for these trials was the occurrence of the welding of contacts. After the test trial, the relay’s coil was disconnected from the power supply and the contacts’ position was tested with an ohmmeter. The low value of the contact resistance indicated that the contacts were welded as they stayed in the close position without the external force provided by the electromagnetic coil. For contacts made of AgNi, welding occurred for three out of four trials, and for those made of AgCdO, welding occurred for each trial. Table 4 presents the mean values of the contact resistance which were calculated on the basis of trials, where there were no cases of welding of contacts. Therefore, for contacts made of AgCdO, there is a lack of calculated values, and for AgNi, it refers only to a single measurement. The welding observed is related to contact bouncing that occurred during the test. For seven out of eight test trials of AgNi, a bounce appeared, for AgSnO_2_ it occurred in five, for AgSnO_2_ P it occurred in six, and for AgCdO it occurred in four tests. As AgNi and AgCdO are less resistant to contact welding, the phenomenon occurred in them and the contact made of AgSnO_2_ and AgSnO_2_ P remained resistant. Studies have shown [25] that contact welding occurs only when the switching phase is π/2 and a bounce occurs. However, some materials, like AgSnO_2_ and AgSnO_2_ P, are immune to contact welding at this current level, as compared to AgNi and AgCdO.

**Figure 6:**
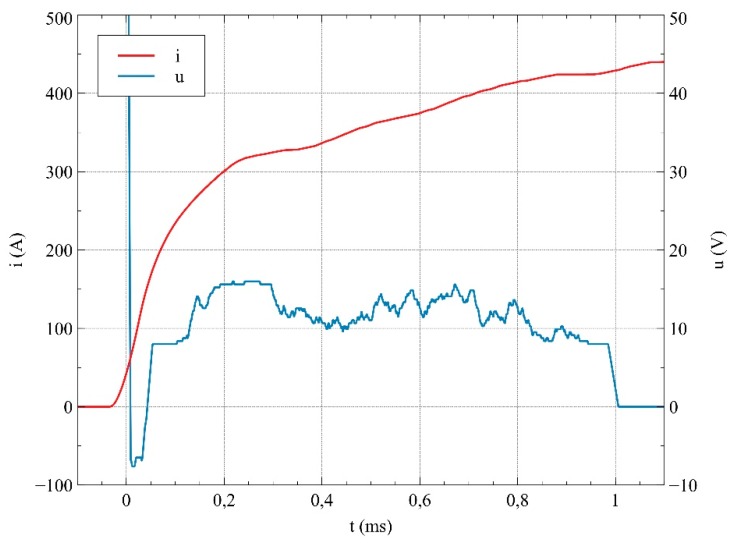
Current and voltage waveforms for a selected test with visible contact bounce.

An example of the switching oscillogram with the registered contact bounce is presented in Figure 6. It can be observed that, in the period of time between around 45 μs to 1 ms, the mean value of voltage between relay contacts equaled approximately 20 V (i.e., the same as the voltage drop of the electric arc in the air). The voltage (8 V) remaining after 1 ms was recorded after switching off the circuit, and it is believed that it was due to the working mechanism of the differential probe used.

The question is the following: why do the same types of relays for the same values of expected short-circuit current behave differently for different phases of switching the current? The answer comes from the combination of two mechanisms: electro-dynamic forces [26] having an impact during the flow of current of significant values and the bounce which results from the impact of two contacts against each other. While switching the circuit at zero phase of voltage, the electro-dynamic force reaches its maximum value after a time of 5 ms. Thus, these two mechanisms do not overlap in time, the resultant opening force of contacts is lower than the clamping force, and there is no contact bounce. For the second case, the electro-dynamic force reaches the maximum which is synchronized with the value of a short-circuit current, that is, just after switching on the circuit. It leads to the overlapping of these two mechanisms in time and, as the consequence, it leads to the contact bounce. During this bounce, at the ignition of an electric arc, there is a pressure increase of the plasma which is located between the relay contacts, which enhances their opening effect. The ignited electric arc leads to a strong, local heating of the contact surface. Its temperature may exceed the value of the material’s melting temperature. Closing the contacts in such a case leads to solid metallic welds, that is, the durable welding of contacts. The calculated value of the electrodynamic force is in the range between 0.20 N and 0.25 N, with the lower value referring to AgNi and the higher value to AgCdO; for AgSnO_2_ and AgSnO_2_ P, the value is equal to 0.21 N. As the value is lower than the nominal contact force shown in Table 2, it is clear that without the contact bounce occurring during the making process, the contact would not have been welded. It is believed that, for welded contacts, the measurement of contact resistance is unjustified; thus, there is no record of such trials in Table 4. For contacts made of AgSnO_2_ and AgSnO_2_ P, there is no welding also for trials with contact bounce. For AgSnO_2_ P, the value of the mean contact resistance after the trial decreased analogically as for trials of switching at zero value of supply voltage. Only for trials of contacts made of AgSnO_2_ was there a slight increase of the mean value of the contact resistance. This increase, however, was calculated only once on the basis of the single trial for which there was an increase of contact resistance. For the remaining three trials, a decrease was observed. For trials resulting in the welding of contacts, there was also the measurement taken of transition resistance. Its value is marginal in comparison to other intended values of resistance.

## 5. Conclusions

The presented results of this research indicate the influence of making a short-circuit current on the value of the contact resistance. This influence depends not only on the contact material which relay contacts are made of, but also on the switching phase of a short-circuit current.

Switching on at the moment when the supply current reaches a zero value leads to a decrease of the value of the contact resistance. The initial value of the contact resistance, after the trial performance of switching on a short-circuit current at zero voltage, decreased significantly. Switching on at the moment when the supply voltage reaches maximal value often leads to the welding of contacts. The value of contact resistance changed for contacts which were not welded, but these changes were not so apparent in comparison with the first discussed cases.

It is worth mentioning that the protection device applied in the testing circuit did not ensure a sufficient level of protection for relay contacts. The protection device should ensure that the protected circuit and all its components are not dysfunctional after a short circuit.

Contacts made of AgNi and AgCdO turned out to be prone to welding. In the range of short-circuit currents up to 320 A, the contacts made of AgSnO_2_ and AgSnO_2_ P were characterized by resistance to welding. These results are consistent with the data presented in the literature [10,11,12,16].

As the electrical contact resistance (ECR) is an important factor in determining the overall relay lifetime reliability, knowledge on how it is influenced by short-circuit current becomes relevant. The ECR value is also a key factor for the design stage of a relay, as it influences its rated current, for example. Future work should include tests with both higher and lower current together with research on material transfer and on contact rivet mass loss.

## Figures and Tables

**Table 1:** Selected properties of the contact materials used in the tests.

Material	Density	Hardness	Thermal Conductivity	Electrical Resistivity
	(kg·m^−3^)	(HB)	(W·K^−1^·m^−1^)	(Ω·m)
AgNi	10,300	50	350	1.84 × 10^−8^
AgSnO_2_/AgSnO_2_ P	9900	70	307	2.04 × 10^−8^
AgCdO	10,200	70	307	2.00 × 10^−8^

**Table 2:** Contact force for different contact materials.

AgNi (N)	AgSnO_2_ (N)	AgSnO_2_ P (N)	AgCdO (N)
0.39	0.33	0.26	0.55

**Table 3:** Mean values of the time periods for short circuits *t_z_*, Joule’s integral *i*^2^*t*, and maximal short-circuit current *i_m_* at switching the short-circuit current.

Protection Device	*t_z_*	*i* ^2^ *t*	*i_m_*
	(ms)	(A^2^s)	(A)
**Relays switched at maximum of supply voltage**			
B16	4.52	448.45	426.18
C16	4.27	442.19	437.71
D16	4.12	458.86	445.71
gG16	4.52	414.08	459.43
**Relays switched at zero of supply voltage**			
B16	6.47	479.85	395.13
C16	6.74	554.66	409.26
D16	5.87	612.24	439.38
gG16	5.81	389.85	443.49

**Table 4:** Mean values of the contact resistance for relays switched on at zero and maximum of supply voltage, before and after the trial: *Ř_b_*, mean value of contact resistance before the trial; *Ř_a_*, mean value of contact resistance after the trial.

Protection Device	*Ř_b_*	*Ř_a_*
	(mΩ)	(mΩ)
**Relays switched at maximum of supply voltage**		
B16	0.5096	0.3309
C16	1.3680	1.4707
D16	1.8909	0.3570
gG16	3.6915	-
**Relays switched at zero of supply voltage**		
B16	1.6857	0.3041
C16	4.1105	0.3817
D16	5.6271	0.2740
gG16	3.7898	0.2615
